# Risk prediction for 30-day mortality among patients with *Clostridium difficile* infections: a retrospective cohort study

**DOI:** 10.1186/s13756-019-0642-z

**Published:** 2019-11-12

**Authors:** Hsiu-Yin Chiang, Han-Chun Huang, Chih-Wei Chung, Yi-Chun Yeh, Yi-Chin Chen, Ni Tien, Hsiu-Shan Lin, Mao-Wang Ho, Chin-Chi Kuo

**Affiliations:** 1 Big Data Center, China Medical University Hospital, Taichung, 404 Taiwan; 2 Department of Medical Research, Department of Internal Medicine, China Medical University Hospital, Taichung, 404 Taiwan; 3 Department of Laboratory Medicine, China Medical University Hospital, Taichung, 404 Taiwan; 4 Division of Infectious Diseases, Department of Internal Medicine, China Medical University Hospital, Taichung, 404 Taiwan; 5 Kidney Institute and Division of Nephrology, Department of Internal Medicine, China Medical University Hospital, Taichung, 404 Taiwan

**Keywords:** *Clostridium difficile infection*, Mortality, ICU stay, Glucose, BUN-to-SCr ratio

## Abstract

**Background:**

Current guidelines have unsatisfied performance in predicting severe outcomes after *Clostridium difficile* infection (CDI). Our objectives were to develop a risk prediction model for 30-day mortality and to examine its performance among inpatients with CDI.

**Methods:**

This retrospective cohort study was conducted at China Medical University Hospital, a 2111-bed tertiary medical center in central Taiwan. We included adult inpatients who had a first positive *C. difficile* culture or toxin assay and had diarrhea as the study population. The main exposure of interest was the biochemical profiles of white blood cell count, serum creatinine (SCr), estimated glomerular filtration rate, blood urea nitrogen (BUN), serum albumin, and glucose. The primary outcome was the 30-day all-cause mortality and the secondary outcome was the length of stay in the intensive care units (ICU) following CDI. A multivariable Cox model and a logistic regression model were developed using clinically relevant and statistically significant variables for 30-day mortality and for length of ICU stay, respectively. A risk scoring system was established by standardizing the coefficients. We compared the performance of our models and the guidelines.

**Results:**

Of 401 patients, 23.4% died within 30 days. In the multivariable model, malignancy (hazard ratio [HR] = 1.95), ≥ 1.5-fold rise in SCr (HR = 2.27), BUN-to-SCr ratio > 20 (HR = 2.04), and increased glucose (≥ 193 vs < 142 mg/dL, HR = 2.18) were significant predictors of 30-day mortality. For patients who survived the first 30 days of CDI, BUN-to-SCr ratio > 20 (Odds ratio [OR] = 4.01) was the only significant predictor for prolonged (> 9 days) length of ICU stay following CDI. The Harrell’s *c* statistic of our Cox model for 30-day mortality (0.727) was significantly superior to those of SHEA-IDSA 2010 (0.645), SHEA-IDSA 2018 (0.591), and ECSMID (0.650). Similarly, the conventional *c* statistic of our logistic regression model for prolonged ICU stay (0.737) was significantly superior to that of the guidelines (SHEA-IDSA 2010, *c* = 0.600; SHEA-IDSA 2018, *c* = 0.634; ESCMID, *c* = 0.645). Our risk prediction scoring system for 30-day mortality correctly reclassified 20.7, 32.1, and 47.9% of patients, respectively.

**Conclusions:**

Our model that included novel biomarkers of BUN-to-SCr ratio and glucose have a higher predictive performance of 30-day mortality and prolonged ICU stay following CDI than do the guidelines.

## Background

*Clostridium difficile* infection (CDI) is a critical healthcare-associated infection and accounts for 20–30% of antibiotic-associated diarrhea [1, 2]. The *Antibiotic Resistance Threats in the United States* report prioritized *C. difficile* as an urgent threat because it spreads rapidly and is naturally resistant to many antimicrobials used to treat other infections [3].

Predicting patients with CDI who are at risk of developing severe complications can guide appropriate treatment and follow-up, and in turn, prevent adverse outcomes [4, 5]. SHEA-IDSA 2010 and SHEA-IDSA 2018 clinical practice guidelines for treating CDI recommend using vancomycin or fidaxomicin to treat initial severe CDI [6, 7]. Two published studies provided evidence that, as high as 31.2–38% of severe CDI and 56–65% of severe-complicate CDI were under-treated [4, 5]. Compared with patients who were treated appropriately, those who were under-treated (according to SHEA-IDSA 2010 guideline) [6] were more likely to have adverse outcomes of all-cause mortality (Crowell’s: 7.2% vs 15.0%; Patel’s: 12.9% vs 43.5%), CDI-related mortality (Crowell’s: 3.8% vs 7.7%; Patel’s: 8.9% vs 21.7%), prolonged CDI-related hospital length of stay (Crowell’s: mean 7.5 days vs 9.4 days), or CDI-related ICU transfer (Patel’s: 4.8% vs 17.4%) [4, 5]. When patients were stratified by severity (defined by SHEA-IDSA 2010 guideline) [6], patients with severe CDI who were under-treated experienced more complications than those who were appropriately treated (death: 20% vs 18.5% for severe CDI; ICU transfer: 20% vs 7.4% for severe CDI), although these findings were not statistically significant [5]. Therefore, identification of potentially severe cases of CDI could provide evidence for appropriate treatment and lead to better patient outcomes.

Conventionally, marked leukocytosis, acute rise in serum creatinine (SCr), hypoalbuminemia, and older age are considered to be prognostic factors of severe complications (ie, intensive care unit [ICU] admission, colectomy, or death), according to guidelines developed by the Society for Healthcare Epidemiology of America and the Infectious Disease Society of America (SHEA-IDSA) in 2010 and 2018 and guidelines developed by the European Society of Clinical Microbiology and Infectious Diseases (ESCMID) in 2014 [6–8]. Although these indicators reasonably represent the underlying interactions between infection, immune–inflammatory responses, and malnutrition, their performance in predicting CDI severity is unsatisfactory [7, 9].

Other severity indices that included comorbidities (eg, malignancy and renal disease) [10, 11], symptoms (eg, fever, hypotension, septic shock, pseudomembranous colitis, and ascites) [12–14], or antibiotic utilization [15, 16] as severity predictors have been reported to improve risk assessment of CDI severity in inpatients or ICU settings [17]. However, subjective measures, different outcomes (ie, mortality, colectomy, ICU admission, recurrence, or cure rate), and inconsistent CDI diagnostic criteria (eg, without information of diarrhea status) [9, 18], compromise the comparability and generalizability of the previous findings [17]. From a pathophysiological perspective, dehydration, a warning sign of severe diarrhea and subsequent hemodynamic instability, should certainly be considered but has never been evaluated as a risk predictor for severe CDI. Blood urea nitrogen (BUN)-to-SCr ratio, which can quantify dehydration and distinguish pre renal kidney injury from intrinsic kidney disease, is a potential predictor for severe CDI.

To address the aforementioned gaps, we conducted this study to develop a new risk prediction model incorporating comorbidities, markers of infection, renal function, dehydration, and serum glucose to predict the risk of 30-day mortality, and to compare the predictive performance of our model and existing guidelines among adult patients with symptomatic CDI.

## Methods

### Data source

This retrospective cohort study was conducted at China Medical University Hospital (CMUH), a 2111-bed tertiary medical center in Taiwan. The data source was the CMUH–Clinical Research Data Repository (CRDR), which accumulates the single unified views of 2,660,472 patients who had sought care at CMUH between 2003 and 2016. The Institutional Review Board of CMUH approved this study (105-REC3–068 & 107-REC2–016).

### Study population

**Fig. 1:**
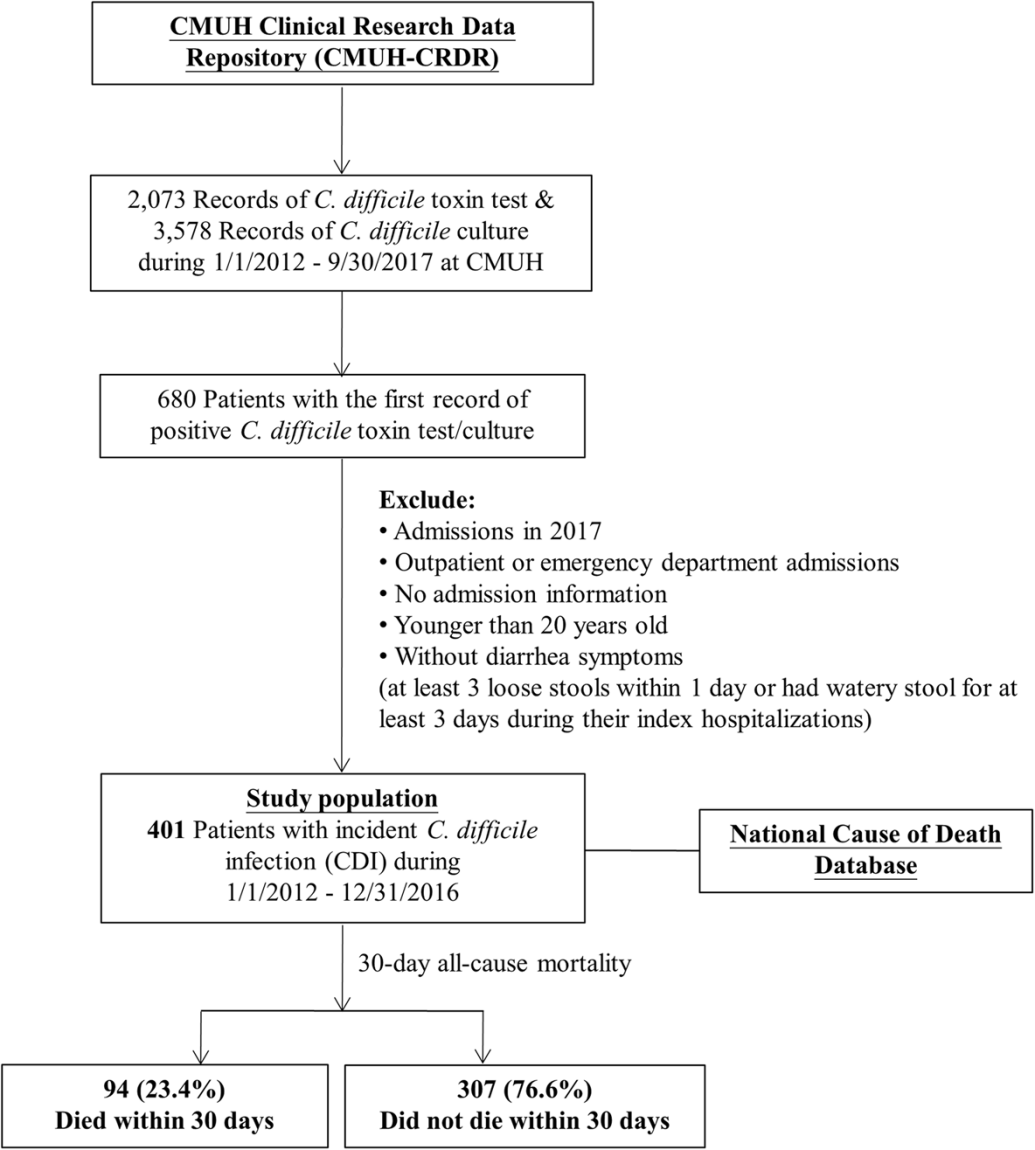
Flowchart of the selection process of the study population. Abbreviation: CMUH, China Medical University Hospital

Our study included all patients who had first-time positive results of *C. difficile* toxin assay or culture at CMUH between January 1, 2012, and December 31, 2016. The index date was the date when the specimen of positive *C. difficile* result was obtained. We excluded patients who 1) were aged younger than 20 years, 2) were not admitted, or 3) did not have diarrhea (at least 3 loose stools per day or loose stools for at least 3 days during hospitalization) [14, 19]. Data were pulled from the CMUH–CRDR, except for diarrhea status, which was manually reviewed using medical records. The mortality data were obtained by linking to the National Cause of Death Database. Our study population comprised 401 adult inpatients who had incident symptomatic CDI (Fig. 1).

### Covariables and outcomes

*C. difficile* testing was performed in inpatients at physicians’ discretion, except that universal screening was performed in patients who were admitted to the medical ICU during the period between January 1, 2014, and February 28, 2015. Methods of *C. difficile* testing were presented in Additional file 1: Methods. The main exposure of interest was the biochemical profiles of white blood cell count (WBC), SCr, estimated glomerular filtration rate (eGFR) through CKD-EPI equation [20], BUN, serum albumin, and glucose that were measured within − 30 to − 3 days of the index date (baseline) or measured within ±3 days of the index date (index). The definitions of variables are listed in Additional file 1**:** Figure S1. The primary outcome of interest was the 30-day all-cause mortality following the index CDI and the secondary outcome of interest was the length of ICU stay following CDI (for patients who survived the first 30 days of CDI).

### Severity predictors from guidelines

Previous guidelines have provided certain severity predictors for identifying severe cases of CDI. The SHEA-IDSA 2010 criteria for a severe CDI are outlined as follows: having a WBC of ≥15,000 cells/μL or a 1.5-fold relative increase in SCr (compared with premorbid level) [6]. The SHEA-IDSA 2018 criteria are presented as follows: having a WBC of ≥15,000 cells/μL or an index SCr of ≥1.5 mg/dL (133 μM) [7]. The ESCMID 2014 criteria are outlined as follows: being aged ≥65 years, having a WBC of ≥15,000 cells/μL, a serum albumin level of < 3.0 g/dL, or a SCr level of ≥ 1.5 mg/dL (133 μM) or a 1.5-fold relative increase in SCr [8].

### Statistical analyses

Continuous variables are presented as medians and interquartile ranges (IQR) and were analyzed using the Wilcoxon rank-sum test. Categorical variables are presented as frequency and proportions (%) and were analyzed using a chi-square test or Fisher’s exact test. All analyses were 2 sided, and the significance level was set to 0.05.

To develop the risk prediction model for 30-day mortality, variables that were significantly associated with 30-day mortality in the univariable analyses (i.e., *P* < 0.05) and that were clinically relevant were considered in the multivariable Cox proportional hazard model. We categorized the included variables in the multivariable model in the subsequent risk score development. Because of the high proportion of missing values for laboratory tests, we performed multiple imputation using an iterative Markov chain Monte Carlo procedure with 20 imputations and 100 iterations [21]. We used the original data and the data from multiple imputations in separate Cox models for 30-day mortality and in separate logistic regression models for prolonged (> 9 days) post-CDI length of ICU stay. We compared the performance of our risk prediction model with that of the guidelines by using discrimination measure of Harrell’s *c* statistic for Cox models [22] or conventional *c* statistic for logistic regression models.

To develop the risk prediction scores, we assigned each independent variable a risk point, which was derived by dividing the beta regression coefficient of each variable by the smallest absolute coefficient and rounding off the quotient to the nearest integer [23]. A severity score was calculated for each patient by summing up the risk points corresponding to the risk factors. We then divided the study population into 2 groups on the basis of their severity scores (< 29 vs ≥29). We compared the performance of our risk prediction scoring system with that of the guidelines by using the reclassification measure of net reclassification index (NRI) [24].

Statistical analysis was performed using SAS version 9.4 (SAS Institute Inc., Cary, NC, USA) and R version 3.0.2 (R Foundation for Statistical Computing, Vienna, Austria) software. All analyses were 2 sided, and the significance level was set to 0.05.

## Results

### Description of patients with *C. difficile* infections

Of 401 inpatients with CDI, the mean age was 68.2 years, 59.1% were men, and 59.3% had documented fever (Table 1). Positive *C. difficile* toxin test results were detected in 54.1% of the patients and positive culture results were found in the remaining patients. The median hospital stay was 25 days, 52.9% were admitted to the ICU, and 23.4% died within 30 days after the index CDI.

**Table 1:** Baseline demographic and clinical characteristics of adult inpatients with *Clostridium difficile* infections (CDI)

Variables^a^	Total (*N* = 401)		30-Day Mortality				*p*-value^a^
			Died (*N* = 94)		Did not die (N = 307)		
Age at index date, years							
Mean (standard deviation)	68.2	15.8	72.5	13.2	66.9	16.4	0.001^b^
≥ 65 years old	234	58.4%	63	67.0%	171	55.7%	0.051
Male	237	59.1%	59	62.8%	178	58.0%	0.409
Comorbidity within 1 year prior^c^							
Diabetes mellitus	201	50.1%	50	53.2%	151	49.2%	0.497
Renal disease	158	39.4%	35	37.2%	123	40.1%	0.623
Inflammatory bowel disease	6	1.5%	1	1.1%	5	1.6%	0.372^b^
Malignancy	154	38.4%	50	53.2%	104	33.9%	0.001
Hospital admission within 90 days prior	182	45.4%	49	52.1%	133	43.3%	0.134
Antibiotic use within 30 days prior							
Cephalosporins	170	42.4%	39	41.5%	131	42.7%	0.839
Fluoroquinolones	81	20.2%	20	21.3%	61	19.9%	0.766
Carbapenems	100	24.9%	25	26.6%	75	24.4%	0.671
Anti-peptic ulcer agents^d^	296	73.8%	68	72.3%	228	74.3%	0.710
APACHE II score prior to CDI^e^	15.0	(10.0, 20.0)	18.0	(11.0, 21.0)	15.0	(10.0, 20.0)	0.036
Fever (≥38 °C) at index CDI	223	59.3%	59	70.2%	164	56.2%	0.021
Anti-diarrhea medications^f^	225	56.1%	51	54.3%	174	56.7%	0.679
Stool routine							
Presence of mucus	52	15.6%	10	14.1%	42	16.0%	0.689
Positive for red blood cell	121	36.3%	26	36.6%	95	36.3%	0.955
Positive for WBC	115	34.5%	24	33.8%	91	34.7%	0.884
*C. difficile* toxin or culture							
Toxin test^g^	217	54.1%	49	52.1%	168	54.7%	0.659
Culture only	184	45.9%	45	47.9%	139	45.3%	
Biochemical profiles at index CDI^h^							
White blood cell count (WBC), cells/mm^3^	12,000	(8300, 17,100)	13,700	(8600, 20,900)	11,700	(8100, 16,400)	0.014
First tertile: < 9440	129	33.0%	27	28.7%	102	34.3%	0.017
Second tertile: 9440 to < 14,600	126	32.2%	23	24.5%	103	34.7%	
Third tertile: ≥14,600	136	34.8%	44	46.8%	92	31.0%	
WBC > 15,000	126	32.2%	41	43.6%	85	28.6%	0.007
Serum creatinine (SCr), mg/dL							
Premorbid SCr^i^	1.01	(0.67, 2.05)	0.97	(0.60, 1.56)	1.03	(0.69, 2.30)	0.392
Index SCr	1.41	(0.81, 3.74)	1.64	(0.90, 3.66)	1.34	(0.76, 3.82)	0.224
Rise in SCr level							
≥ 1.5-fold	113	34.9%	46	55.4%	67	27.8%	< 0.001
≥ 1.5045 mg/dL	56	17.3%	19	22.9%	37	15.4%	0.1172
≥ 1.5-fold or ≥ 1.5045 mg/dL	123	38.0%	48	57.8%	75	31.1%	< 0.001
eGFR (CKD-EPI)^j^, ml/min/1.73m^2^	51.4	(17.3, 91.0)	47.3	(16.7, 88.3)	52.5	(17.6, 92.5)	0.623
BUN, mg/dL	29.0	(14.0, 60.0)	41.0	(21.0, 85.0)	25.0	(13.0, 52.5)	< 0.001
First tertile: < 17	110	30.8%	14	16.5%	96	35.3%	0.004
Second tertile: 17 to < 44	121	33.9%	33	38.8%	88	32.4%	
Third tertile: ≥44	126	35.3%	38	44.7%	88	32.4%	
BUN > 26	188	52.7%	55	64.7%	133	48.9%	0.011
BUN-to-SCr ratio	18.8	(12.2, 30.1)	26.1	(17.6, 36.8)	16.8	(11.1, 27.3)	< 0.001
Index BUN-to-SCr > 20	170	47.6%	59	69.4%	111	40.8%	< 0.001
Albumin, g/dL	2.50	(2.20, 2.90)	2.35	(2.00, 2.80)	2.60	(2.20, 3.00)	0.003
Albumin < 2.5	89	44.3%	33	56.9%	56	39.2%	0.022
Albumin < 3	155	77.1%	50	86.2%	105	73.4%	0.051
Serum glucose, mg/dL	162	(129, 222)	192	(151, 232)	158	(127, 208)	0.002
First tertile: < 142	107	32.7%	13	16.9%	94	37.6%	0.001
Second tertile: 142 to < 193	108	33.0%	26	33.8%	82	32.8%	
Third tertile: ≥193	112	34.3%	38	49.4%	74	29.6%	

Abbreviations: *APACHE* Acute Physiology and Chronic Health Evaluation, *BUN* blood urea nitrogen level, *CDI C. difficile* infections, *CI* confidence interval, *eGFR* estimated Glomerular filtration rate, *HR* hazard ratio, *IQR* interquartile range, *SCr* serum creatinine, *WBC* white blood cell count

^a^Continuous variables were presented as median and IQRs and analyzed using Wilcoxon rank-sum test, if not otherwise indicated. Categorical variables were presented as frequency and proportion (%) and analyzed using chi-square test, if not otherwise indicated. *P*-values that were < 0.05 are shown in bold.

^b^Mean age and the proportion of inflammatory bowel disease were analyzed using two-sample t-test and Fisher’s exact test, respectively.

^c^Diabetes mellitus was defined according to the patients’ ICD-9-CM diagnosis codes and the use of glucose-lowering agents. Renal disease, inflammatory bowel disease, and malignancy were defined using the ICD-9-CM diagnosis codes.

^d^Use of anti-peptic ulcer agents of proton-pump inhibitors and histamine-2 receptor antagonists within 0 to 14 days of the index CDI.

^e^APACHE II score was only available for patients admitted to intensive care units (*N* = 211).

^f^Use of anti-diarrhea medications or probiotics within 0 to 14 days of the index CDI.

^g^Included 158 patients (39.4%) with positive toxin genes test and 59 patients (14.7%) with positive C. difficile toxin enzyme immunoassay test.

^h^We obtained the maximum WBC, maximum index SCr, closest BUN, minimum albumin, and closest glucose values that were measured within −3 to + 3 days of the index CDI.

^i^For premorbid SCr, we obtained the minimum SCr that were measured within − 30 to −4 days of the index CDI.

^j^eGFR was estimated by CKD-EPI equation (Levey 2009).

### Characteristics associated with 30-day mortality

Patient who died within 30 days following their CDI were more likely to be older (mean age: 72.5 vs 66.9 years), have malignancies (eg, leukemia and lymphoma; 53.2% vs 33.9%), have fever (70.2% vs 56.2%), and have higher Acute Physiology and Chronic Health Evaluation (APACHE) II scores among patients admitted to ICUs prior to CDI (median 18 vs 15), compared with those who survived within 30 days (Table 1).

The biochemical profiles significantly differed between patients who died and those who survived, except for the baseline eGFR. Patients who died had higher levels of WBC (median 13,700 vs 11,700 cells/μL), an increased likelihood of having an SCr level 1.5-fold higher than their premorbid level (55.4% vs 27.8%), higher levels of BUN (median 41.0 vs 25.0 mg/dL), an increased likelihood of having a BUN-to-SCr ratio of > 20 (69.4% vs 40.8%), lower levels of albumin (median 2.35 vs 2.60 g/dL), and higher levels of glucose (median 192 vs 158 mg/dL).

### Risk prediction model for 30-day mortality

**Fig. 2:**
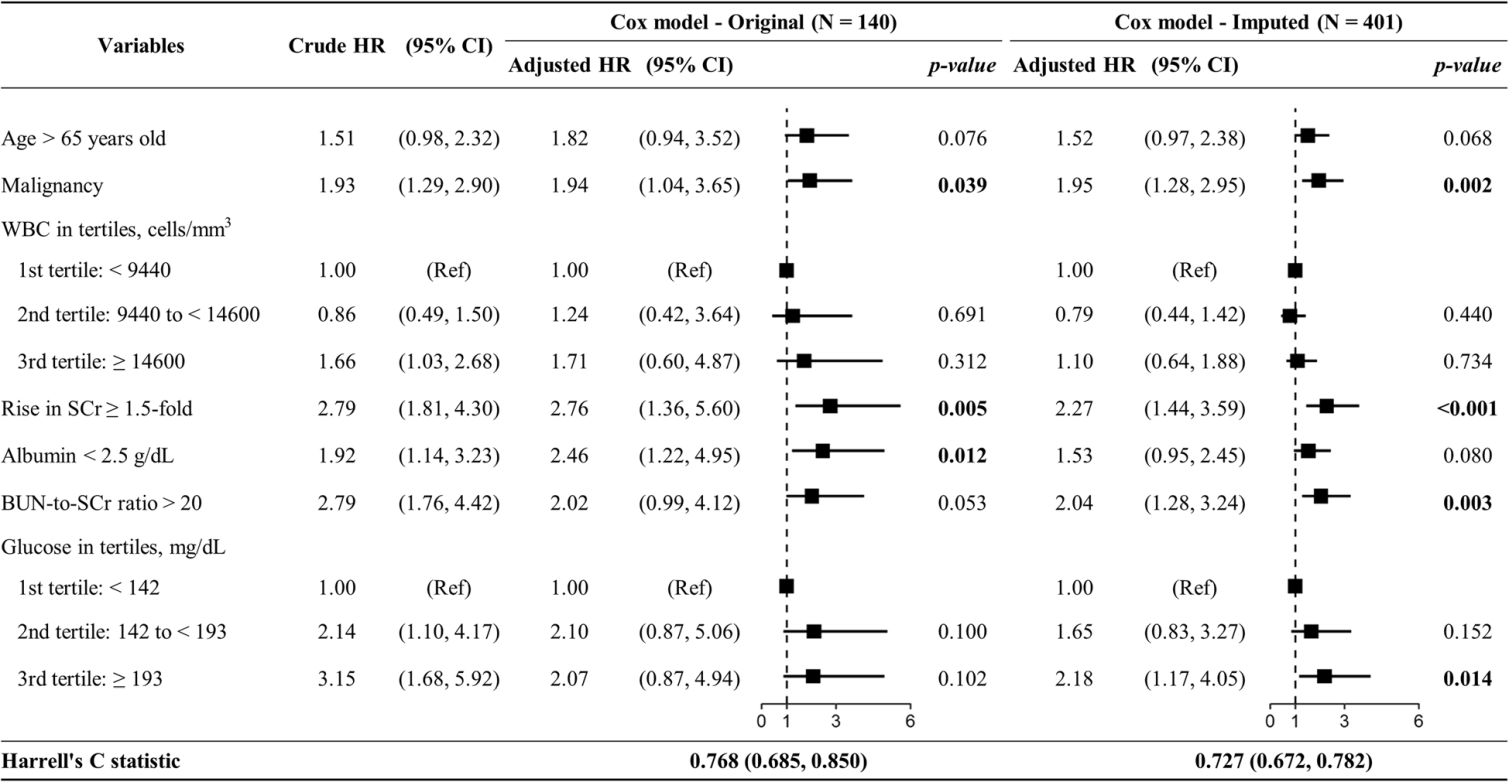
Risk prediction model for 30-day mortality among adult inpatients with *C. difficile* infections. We included variables in a Cox proportional hazard model and evaluated the discrimination performance using Harrell’s *c* statistic. Abbreviations: BUN, blood urea nitrogen; CI, confidence interval; HR, hazard ratio; Ref, reference; SCr, serum creatinine; WBC, white blood cell count

To develop the risk prediction model, we included age > 65 years, malignancy history, index WBC in tertiles, 1.5-fold rise in SCr, albumin < 2.5 g/dL, BUN-to-SCr ratio > 20, and glucose in tertiles in a Cox model (Fig. 2). The results obtained for the original and imputed data were similar. Malignancy (hazard ratio [HR] = 1.95; 95% confidence interval [CI] = 1.28, 2.95), rise in SCr (HR = 2.27, 95% CI = 1.44, 3.95), BUN-to-SCr ratio > 20 (HR = 2.04, 95% CI = 1.28, 3.24), and glucose level ≥ 193 mg/dL (reference: < 142 mg/dL, HR = 2.18, 95% CI = 1.17, 4.05) were significantly associated with 30-day mortality when imputed data were used. The discrimination performance of our model (Harrell’s *c* statistic = 0.727; 95% CI = 0.672, 0.782) was significantly superior to that of the model using severity indicators stated in the SHEA-IDSA 2010 (*c* statistic = 0.645; 95% CI = 0.588, 0.702), SHEA-IDSA 2018 (*c* statistic = 0.591; 95% CI = 0.537, 0.644), and the ESCMID guidelines (*c* statistic = 0.650; 95% CI = 0.594, 0.711) (Table 2).

**Table 2:** Discrimination performance of published guidelines for 30-day mortality among adult inpatients with *C. difficile* infections^a^

Variables	SHEA-IDSA (2010)^b^				SHEA-IDSA (2018)^b^				ESCMID (2014)^c^			
	Original (*N* = 322)		Imputed (N = 401)		Original (*N* = 374)		Imputed (*N* = 401)		Original (*N* = 165)		Imputed (N = 401)	
	aHR (95% CI)	*p*-value	aHR (95% CI)	*p*-value	aHR (95% CI)	*p*-value	aHR (95% CI)	*p*-value	aHR (95% CI)	*p*-value	aHR (95% CI)	*p*-value
Age > 65 years old	–	–	–	–	–	–	–	–	1.44 (0.84, 2.47)	0.185	1.41 (0.91, 2.17)	0.124
WBC > 15,000 cells/mm^3^	1.44 (0.92, 2.24)	0.110	1.43 (0.92, 2.24)	0.113	1.72 (1.13, 2.61)	0.012	1.69 (1.11, 2.56)	0.013	1.91 (1.12, 3.27)	0.018	1.51 (1.00, 2.29)	0.053
Rise in SCr ≥1.5-fold	2.55 (1.64, 3.98)	< 0.0001	2.58 (1.65, 4.03)	< 0.0001	–	–	–	–	–	–	–	–
SCr ≥1.5045 mg/dL	–	–	–	–	1.27 (0.84, 1.93)	0.265	1.35 (0.89, 2.04)	0.157	–	–	–	–
Rise in SCr ≥1.5-fold or ≥ 1.5045 mg/dL	–	–	–	–	–	–	–	–	1.40 (0.77, 2.54)	0.268	2.23 (1.37, 3.61)	0.001
Albumin < 3 g/dL	–	–	–	–	–	–	–	–	1.93 (0.91, 4.08)	0.085	1.60 (0.83, 3.10)	0.159
*c* statistic (95% CI)	0.644 (0.587, 0.701)		0.645 (0.588, 0.702)^d^		0.587 (0.533, 0.641)		0.591 (0.537, 0.644)^d^		0.640 (0.571, 0.709)		0.650 (0.594, 0.706)^d^	

Abbreviations: *aHR* adjusted hazard ratio, *CDI C. difficile* infections, *CI* confidence interval, *SCr* serum creatinine, *WBC* white blood cell count

^a^We included the variables in separate Cox proportional hazard models and evaluated the discrimination performance of these models using Harrell’s *c* statistic.

^b^The Society of Hospital Epidemiology of America (SHEA) and the Infectious Disease Society of America (IDSA) jointly published the clinical practice guidelines for CDI in 2010 and updated in 2018 (Cohen 2010; McDonald 2018).

^c^The European Society of Clinical Microbiology and Infectious Diseases (ESCMID) published the treatment guideline for CDI in 2014 (Debast 2014).

^d^The discrimination performance of these models was significantly lower than that of our prediction model (Harrell’s *c* statistic = 0.727; 95% CI = 0.672, 0.782).

### Risk prediction scoring system for 30-day mortality

To develop a risk prediction scoring system, we assigned each risk predictor a risk point (Table 3). Patients with a risk score of ≥29 were considered to be at a higher risk (10% or higher) of 30-day mortality. Compared with the SHEA-IDSA 2010, SHEA-IDSA 2018, and ESCMID guidelines, our scoring system reclassified 20.7, 32.1, and 47.9% of the CDI patients into the correct risk category, respectively (Additional file 1: Table S1).

**Table 3:** Risk prediction model for 30-day mortality among adult inpatients with *C. difficile* infections and the risk points

Variables	Regression coefficients (βs)	Risk point^a^
Age > 65 years old	0.4179	4
Malignancy	0.6656	7
WBC in tertiles, cells/mm^3^		
First tertile: < 9440	Reference	0
Second tertile: 9440 to < 14,600	−0.2299	−2
Third tertile: ≥14,600	0.0930	1
Rise in SCr ≥1.5-fold	0.8212	9
Albumin < 2.5 g/dL	0.4226	5
Glucose in tertiles, mg/dL		
First tertile: < 142	Reference	0
Second tertile: 142 to < 193	0.4993	5
Third tertile: ≥193	0.7784	8
BUN-to-SCr ratio > 20	0.7119	8

Abbreviations: *BUN* blood urea nitrogen, *SCr* serum creatinine, *WBC* white blood cell count

^a^We assigned each variable a risk point by dividing the corresponding regression coefficient by the absolute smallest coefficient (i.e., 0.0930) and rounding it to the nearest integer.

### Risk prediction model for prolonged length of ICU stay following CDI

Of 307 patients who survived the first 30 days following CDI, the mean length of ICU stay following CDI was 9.8 days (median: 0 days; interquartile range, 0–9 days). We used the 3rd quartile (9 days) as the cut-off for prolonged post-CDI length of ICU stay in the multivariable logistic regression analysis.

We evaluated the performance of our risk prediction model in predicting post-CDI length of ICU stay > 9 days (Table 4). BUN-to-SCr ratio was the only significant and strong predictor for prolonged length of ICU stay following CDI (imputed data: adjusted OR, 4.01; 95% CI, 2.19–7.33). The discrimination performance of our prediction model was moderate (imputed data: *c* statistic, 0.737; 95% CI, 0.671–0.804), and was superior to the discrimination performance of SHEA-IDSA 2010 (*c* statistic, 0.600; 95% CI, 0.527–0.673), SHEA-IDSA 2018 (*c* statistic, 0.634; 95% CI, 0.564–0.704), and ESCMID (*c* statistic, 0.645; 95% CI, 0.573–0.718) (Table 5).

**Table 4:** Risk prediction model for prolonged (> 9 days) length of ICU stay following *C. difficile* infections (CDI) among adult inpatients with CDI who survived the first 30 days following CDI (*N* = 307)^a^

Variables	Crude OR (95% CI)	*P*-value	Logistic regression model - Original (*N* = 99)	*P*-value	Logistic regression model - Imputed (*N* = 307)	*P*-value
			Adjusted OR (95% CI)		Adjusted OR (95% CI)	
Age > 65 years old	1.51 (0.88, 2.57)	0.133	1.37 (0.56, 3.36)	0.492	1.28 (0.72, 2.30)	0.400
Malignancy	0.57 (0.32, 1.03)	0.061	0.60 (0.23, 1.60)	0.310	0.54 (0.29, 1.03)	0.061
WBC in tertiles, cells/mm^3^						
1st tertile: < 9440	1.00 (Ref)		1.00 (Ref)		1.00 (Ref)	
2nd tertile: 9440 to < 14,600	1.33 (0.68, 2.61)	0.412	1.17 (0.37, 3.74)	0.786	1.19 (0.57, 2.52)	0.640
3rd tertile: ≥ 14,600	2.33 (1.21, 4.50)	0.012	1.21 (0.36, 4.01)	0.756	1.76 (0.84, 3.69)	0.132
Rise in SCr ≥ 1.5-fold	1.48 (0.79, 2.76)	0.217	2.40 (0.92, 6.26)	0.073	1.34 (0.69, 2.59)	0.393
Albumin < 2.5 g/dL	2.22 (1.08, 4.54)	0.030	1.89 (0.72, 4.95)	0.195	1.36 (0.66, 2.82)	0.400
BUN-to-SCr ratio > 20	4.74 (2.66, 8.46)	< 0.001	3.81 (1.51, 9.58)	0.005	4.01 (2.19, 7.33)	< 0.001
Glucose tertiles, mg/dL						
1st tertile: < 142	1.00 (Ref)		1.00 (Ref)		1.00 (Ref)	
2nd tertile: 142 to < 193	1.61 (0.83, 3.12)	0.161	1.41 (0.46, 4.33)	0.545	1.28 (0.72, 2.30)	0.480
3rd tertile: ≥ 193	1.57 (0.79, 3.11)	0.194	0.87 (0.28, 2.72)	0.812	0.54 (0.29, 1.03)	0.618
*C* statistic (95% CI)			0.741 (0.639, 0.844)		0.737 (0.671, 0.804)	

Abbreviations: *BUN* blood urea nitrogen, *CI* confidence interval, *OR* odds ratio, *Ref* reference, *SCr* serum creatinine, *WBC* white blood cell count

^a^We included variables in a logistic regression model and evaluated the discrimination performance using conventional *c* statistic.

**Table 5:** Discrimination performance of published guidelines for prolonged (> 9 days) length of ICU stay following *C. difficile* infections (CDI) among adult inpatients with CDI who survived the first 30 days following CDI (N = 307)^a^

Variables	SHEA-IDSA (2010)^b^				SHEA-IDSA (2018)^b^				ESCMID (2014)^c^			
	Original (*N* = 239)		Imputed (*N* = 307)		Original (*N* = 283)		Imputed (*N* = 307)		Original (*N* = 143)		Imputed (N = 307)	
	aOR (95% CI)	*p*-value	aOR (95% CI)	*p*-value	aOR (95% CI)	*p*-value	aOR (95% CI)	*p*-value	aOR (95% CI)	*p*-value	aOR (95% CI)	*p*-value
Age > 65 years old	–	–	–	–	–	–	–	–	1.25 (0.60, 2.60)	0.549	1.45 (0.84, 2.51)	0.182
WBC > 15,000 cells/mm^3^	1.96 (1.06, 3.64)	0.033	2.19 (1.25, 3.83)	0.006	2.26 (1.28, 3.99)	0.005	2.10 (1.20, 3.67)	0.009	1.80 (0.84, 3.85)	0.129	2.11 (1.20, 3.70)	0.010
Rise in SCr ≥1.5-fold	1.31 (0.69, 2.49)	0.403	1.30 (0.70, 2.40)	0.411	–	–	–	–	–	–	–	–
SCr ≥1.5045 mg/dL	–	–	–	–	1.91 (1.10, 3.31)	0.022	1.84 (1.07, 3.16)	0.026	–	–	–	–
Rise in SCr ≥1.5-fold or ≥ 1.5045 mg/dL	–	–	–	–	–	–	–	–	2.38 (1.08, 5.23)	0.031	1.74 (0.98, 3.09)	0.057
Albumin < 3 g/dL	–	–	–	–	–	–	–	–	1.74 (0.72, 4.17)	0.219	1.29 (0.58, 2.85)	0.529
*C* statistic (95% CI)	0.591 (0.513, 0.670)		0.600 (0.527, 0.673)^d^		0.643 (0.572, 0.714)		0.634 (0.564, 0.704)^d^		0.657 (0.561, 0.754)		0.645 (0.573, 0.718)^d^	

Abbreviations: *aHR* adjusted hazard ratio, *CDI C. difficile* infections, *CI* confidence interval, *SCr* serum creatinine, *WBC* white blood cell count

^a^We included the variables in separate logistic regression models and evaluated the discrimination performance of these models using conventional *c* statistic.

^b^The Society of Hospital Epidemiology of America (SHEA) and the Infectious Disease Society of America (IDSA) jointly published the clinical practice guidelines for CDI in 2010 and updated in 2018 (Cohen 2010; McDonald 2018).

^c^The European Society of Clinical Microbiology and Infectious Diseases (ESCMID) published the treatment guideline for CDI in 2014 (Debast 2014).

^d^The discrimination performance of these models was significantly lower than that of our prediction model (*c* statistic = 0.737; 95% CI = 0.671, 0.804).

## Discussion

This is the first epidemiological study to investigate predictors for the severe outcome of CDI in Asia [25]. Our risk prediction model included age > 65 years, malignancy history, WBC in tertiles, 1.5-fold rise in SCr, albumin < 2.5 g/dL, BUN-to-SCr ratio > 20, and serum glucose in tertiles, where BUN-to-SCr ratio and glucose have not been indicated in prior studies. Our risk prediction model and risk prediction scoring system performed superior to current guidelines in predicting 30-day mortality and prolonged length of ICU stay following CDI.

Published guidelines in the US (SHEA-IDSA 2010 and 2018) [6, 7] and Europe (ECSMID 2014) [8] and recently developed severity indices, such as the Zar [26], Bauer [19], ATLAS [15], Velazquez-Gomez [14], or Gomez-Simmonds [27] scoring systems, have attempted to establish valid criteria for predicting the severity of CDI. Nonetheless, guidelines are based on expert opinions or systematic reviews and the definitions of severe CDI and its treatment outcomes evaluated in other studies have varied. For example, Zar et al. assessed both cure rate and relapse [26]; Bauer et al. evaluated treatment failure and recurrence [19]; and ATLAS evaluated cure rate, and its findings were validated in another cohort for mortality and colectomy [15, 28]. Increased levels of WBC (≥15,000 or 30,000 cells/μL) and rise in SCr (1.5-fold high than the premorbid level or absolute value of 1.5 mg/dL), which indicate immune reaction and renal function, are the most common markers between the aforementioned severity criteria [6, 7, 15, 19]. Hypoalbuminemia (< 2.5 or < 3 mg/dL), a malnutrition marker, is another commonly marker for severe CDI [8, 14, 26, 27].

Other predictors of poor CDI outcomes that have been reported included: older age [26], systemic antibiotic use, underlying illnesses, altered mental status [14], physical findings (eg, fever, hypotension [14], tachycardia [14], abdominal pain or distention, and septic shock [27]), pseudomembranous colitis [14, 26, 27], ICU admission [14, 26], toxic megacolon, and colectomy [27]. However, ICU admission and CDI-related complications should not be used as prognostic predictors because these events are outcomes of severe CDI.

Our severity predictive model had significantly higher discrimination power than did the existing guidelines in predicting 30-day mortality. Our scoring system reclassified 21% (SHEA-IDSA 2010), 32% (SHEA-IDSA 2018), or 46% (ESCMID) of CDI patients into the correct risk category. Consistent with our findings, Stevens et al. showed that both the SHEA-IDSA 2010 and 2018 criteria had low discrimination power (*c* = 0.582 and 0.587) [9]. Further evaluation of these clinical guidelines in high-quality studies is required, which is also suggested by the latest SHEA-IDSA guidelines [7].

Other severity indices did not evaluate their performance in predicting prolonged length of ICU stay separately from other outcomes, but they used composite measure of mortality, ICU admission, or colectomy [18, 27, 29]. However, ICU admission may not be a reasonable outcome measure because many patients with CDI are already in the ICU at the time of CDI occurrence. In our study, we assessed the secondary outcome of prolonged length of ICU stay following CDI and used 9 days as the cut-off. The 9-day cut-off is the 75th quartile in the distribution in our study population and is also comparable to the length of ICU stay attributable to CDI reported in two prior studies [30, 31]. Zahar et al’s assessed the morbidity and mortality attributable to ICU-acquired CDI and estimated that the increase in the ICU stay due to CDI was 8.0 ± 9.3 days, in comparison to the diarrheic population [30]. Dodek et al. also investigated the attributable ICU and hospital length of stay of ICU-acquired CDI and reported that median ICU days following CDI was 7 days (IQR, 3–14 days) [31]. Therefore, an ICU stay of more than 9 days is a clinically relevant outcome measure for patients with CDI. In addition, our risk prediction model can better identity patients at high risk of prolonged ICU stay following CDI than can the guidelines.

Notably, we identified BUN-to-SCr ratio and serum glucose as strong predictors of 30-day mortality. A BUN-to-SCr ratio of > 20 indicates dehydration and an early stage of kidney injury, which reasonably reflects the severity for CDI patients. In contrast, one previous study of 184 CDI patients did not find any association between a BUN-to-SCr ratio of ≥20 and severe outcomes (defined as any event of ICU admission, colectomy, or death within 30 days) [29]. Moreover, no prior study has found an association between increased baseline glucose level and increased mortality among CDI patients. Our study showed that diabetes at admission was not associated with 30-day mortality, but a serum glucose level of ≥193 mg/dL was (HR = 2.18; 95% CI = 1.17, 4.05). One study including 94 CDI patients identified that diabetes was associated with relapse of CDI (odds ratio [OR] = 2.7; 95% CI = 0.8–9.2) [32]. Another study including 247 CDI patients revealed that diabetes was an independent risk factor for recurrent CDI within 6 months (OR = 3.05; 95% CI = 1.84, 5.03) but that serum glucose level was not (median of 147 mg/dL for recurrent CDI and 146 mg/dL for nonrecurrent CDI) [33]. Blood glucose can influence the host immune-inflammatory response, such as macrophages, and affect the community structure of the gut microbiome, such as changing the ratio of nontoxigenic to toxigenic *C. difficile* [34]. Whether a hyperglycemic status in itself or through modification of a patient’s intestinal microbiome facilitates the growth of *C. difficile* warrants further investigation [34]. Both BUN-to-SCr ratio and serum glucose can be clinically modified and can serve as indicators to measure treatment optimization. Future research should clarify whether modifying these predictors can benefit patients with CDI.

Our study has several limitations. First, due to the retrospective design, the screening and diagnosis of CDI were not based on a standardized research protocol and certain variables of interest had missing values. However, we used extensive data—which were electronic medical records from the well-established CMUH–CRDR, expert adjudication of clinical presentation of CDI, and the National Cause of Death Dataset—, and multiple imputation method [21], to compensate for this limitation. Second, not all patients with CDI received molecular typing of *C. difficile*, which prevented us from differentiating toxigenic versus nontoxigenic strains for all CDI and evaluating the prognostic value of strain virulence. Nonetheless, our proposed risk model provides simple and readily available laboratory markers to triage patients with CDI and lower the action threshold to initiate optimization of fluid status and empirical antibiotic therapy.

## Conclusions

Our proposed risk prediction model and scoring system performs more accurately in identifying potentially severe CDI than do existing guidelines. The newly identified clinical markers, namely BUN-to-SCr ratio and glucose, are readily available and also increase awareness of clinicians to optimize supportive care in patients with CDI. Future research should replicate our study in other populations. The infectious disease community should work toward consensus regarding the definition of severity and treatment response of CDI to support comparability of information and evidence-driven decision making for optimal CDI care.

## Supplementary information

**Additional file 1.** Supplementary method: *Clostridium difficile* testing; **Figure S1.** Time frames for the definition of covarieates; **Table S1.** Net reclassification of risk prediction score and the published guidelines.

## Data Availability

The minimal datasets generated and/or analysed during the current study are available from the corresponding author on reasonable request.

## Abbreviations

APACHE: Acute Physiology and Chronic Health Evaluation
BUN: Blood urea nitrogen
CDI: *Clostridium difficile* infection
CI: Confidence interval
CMUH: China Medical University Hospital
CRDR: Clinical Research Data Repository
eGFR: estimated Glomerular filtration rate
ESCMID: The European Society of Clinical Microbiology and Infectious Diseases
HR: Hazard ratio
ICU: Intensive care unit
IQR: Interquartile range
NRI: Net reclassification index
SCr: Serum creatinine
SHEA-IDSA: The Society for Healthcare Epidemiology of America and The Infectious Diseases Society of America
WBC: White blood cell
