# Supplementary material for: Risk prediction for 30-day mortality among patients with Clostridium difficile infections: a retrospective cohort study
Source: Antimicrob Resist Infect Control. 2019 Nov 12;8:175. doi: 10.1186/s13756-019-0642-z (PMC6852910; doi:10.1186/s13756-019-0642-z)
Supplement: Supplementary file 1 — Additional file 1. Supplementary method: Clostridium difficile testing; Figure S1. Time frames for the definition of covarieates; Table S1. Net reclassification of risk prediction score and the published guidelines. [file 13756_2019_642_MOESM1_ESM.docx]

**Additional file 1: Methods.** *C. difficile* Testing.

Stool specimens were cultured anaerobically on cycloserine, cefoxitin, fructose, and egg yolk agar (CCFA) medium. The isolates were identified through MALDI-TOF mass spectrometry (Bruker Daltonics, Germany) using MALDI BIOTYPER 2.0 software (Bruker Daltonics, Billerica, MA, USA). For C. difficile toxin tests, we used the GeneXpert Dx system and Xpert C. difficile/Epi reagent (Cepheid, Sunnyvale, CA, USA) to identify C. difficile toxin genes through a real-time reverse transcription polymerase chain reaction assay, or we used the toxin enzyme immunoassay to detect the presence of toxins A and B (DUO Toxin A + B; VEDA.LAB, France).

**Additional file 1: Figure S1.** Time frames for the definition of covariables. Abbreviation: CDI, *Clostridium difficile* infection.

**
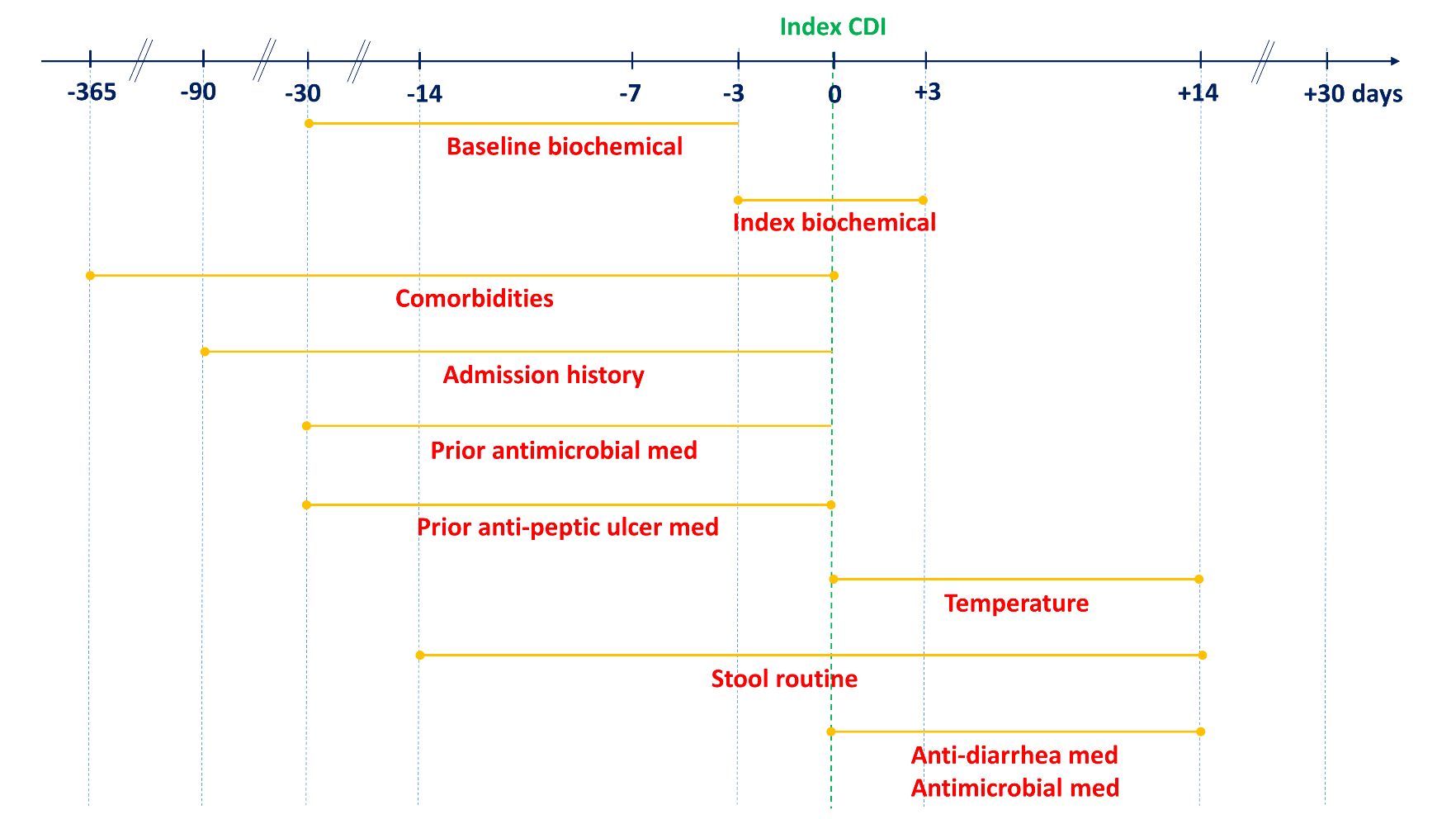
**

**Additional file 1: Table S1.** Net re-classification of risk prediction score and the published guidelines.

1. **SHEA-IDSA (2010) vs. Risk Prediction Score**

|  | **Died within 30 days (N = 41)** | | | |  | **Did not die within 30 days (N = 99)** | | | |
| --- | --- | --- | --- | --- | --- | --- | --- | --- | --- |
|  |  | **Risk Prediction Score^a^** | | |  |  | **Risk Prediction Score^a^** | | |
|  |  | Low risk | High risk | Total |  |  | Low risk | High risk | Total |
| **SHEA- IDSA (2010)^b^** | Low risk | 8 | 1 | 9 |  | Low risk | 48 | 0 | 48 |
|  | High risk | 14 | 18 | 32 |  | High risk | 42 | 9 | 51 |
|  | Total | 22 | 19 | 41 |  | Total | 90 | 9 | 99 |
|  |  |  |  |  |  |  |  |  |  |
|  |  |  |  | **Died** | | **Did not die** |  |  |  |
|  |  | Correct reclassification | | 1 | | 42 |  |  |  |
|  |  | Incorrect reclassification | | 14 | | 0 |  |  |  |
|  |  | Net reclassification | | -13 | | 42 |  |  |  |
|  |  | **Additive NRI** | | 11 | | |  |  |  |
|  |  | **Absolute NRI** | | 20.7% | | |  |  |  |

1. **SHEA-IDSA (2018) vs. Risk Prediction Scores**

|  | **Died within 30 days (N = 41)** | | | |  | **Did not die within 30 days (N = 99)** | | | |
| --- | --- | --- | --- | --- | --- | --- | --- | --- | --- |
|  |  | **Risk Prediction Score^a^** | | |  |  | **Risk Prediction Score^a^** | | |
|  |  | Low risk | High risk | Total |  |  | Low risk | High risk | Total |
| **SHEA- IDSA (2018)^b^** | Low risk | 5 | 2 | 7 |  | Low risk | 28 | 2 | 30 |
|  | High risk | 17 | 17 | 34 |  | High risk | 62 | 7 | 69 |
|  | Total | 22 | 19 | 41 |  | Total | 90 | 9 | 99 |
|  |  |  |  |  |  |  |  |  |  |
|  |  |  |  | **Died** | | **Did not die** |  |  |  |
|  |  | Correct reclassification | | 2 | | 62 |  |  |  |
|  |  | Incorrect reclassification | | 17 | | 2 |  |  |  |
|  |  | Net reclassification | | -15 | | 60 |  |  |  |
|  |  | **Additive NRI** | | 24 | | |  |  |  |
|  |  | **Absolute NRI** | | 32.1% | | |  |  |  |

1. **ESCMID (2014) vs. Risk Prediction Scores**

|  | **Died within 30 days (N = 41)** | | | |  | **Did not die within 30 days (N = 99)** | | | |
| --- | --- | --- | --- | --- | --- | --- | --- | --- | --- |
|  |  | **Risk Prediction Score^a^** | | |  |  | **Risk Prediction Score^a^** | | |
|  |  | Low risk | High risk | Total |  |  | Low risk | High risk | Total |
| **ESCMID (2014)^b^** | Low risk | 1 | 0 | 1 |  | Low risk | 2 | 0 | 2 |
|  | High risk | 21 | 19 | 40 |  | High risk | 88 | 9 | 97 |
|  | Total | 22 | 19 | 41 |  | Total | 90 | 9 | 99 |
|  |  |  |  |  |  |  |  |  |  |
|  |  |  |  | **Died** | | **Did not die** |  |  |  |
|  |  | Correct reclassification | | 0 | | 88 |  |  |  |
|  |  | Incorrect reclassification | | 21 | | 0 |  |  |  |
|  |  | Net reclassification | | -21 | | 88 |  |  |  |
|  |  | **Additive NRI** | | 38 | | |  |  |  |
|  |  | **Absolute NRI** | | 47.9% | | |  |  |  |

Abbreviations: NRI, net reclassification index.

^a.^ Patient with a risk prediction score of 29 or higher was considered to be at high risk of 30-day mortality.

^b.^ The Society of Hospital Epidemiology of America (SHEA) and the Infectious Disease Society of America (IDSA) jointly published the clinical practice guidelines for CDI in 2010 and updated in 2018. (Cohen 2010; McDonald 2018)

^c.^ The European Society of Clinical Microbiology and Infectious Diseases (ESCMID) published the treatment guideline for CDI in 2014. (Debast 2014)
